# A minimal hybridization capture approach for the parallel enrichment and cost-effective detection of ancient human pathogens

**DOI:** 10.1016/j.isci.2026.116625

**Published:** 2026-08-20

**Authors:** Arthur Kocher, Andaine Seguin-Orlando, Pierre Clavel, Guillaume Louvel, Richard Jonvel, Stéfan Tzortzis, Michel Signoli, Caroline Costedoat, Ludovic Orlando

**Affiliations:** 1 Centre d’Anthropobiologie et de Génomique de Toulouse (CAGT), CNRS UMR 5288, Université de Toulouse, 37 Allées Jules Guesde, 31000 Toulouse, France; 2 Amiens Métropole Service Archéologie Préventive, 2 rue Colbert, 80000 Amiens, France; 3 Service Régional de l’Archéologie, 21 allée Claude Forbin, 13100 Aix-en-Provence, France; 4 Aix-Marseille Université, CNRS, EFS, ADES, 13005 Marseille, France

**Keywords:** cellular neuroscience, molecular neuroscience

## Abstract

The preservation of ancient DNA in archaeological remains enables the identification of past disease agents. However, pathogen DNA is typically highly diluted by host and environmental DNA, limiting detection. Here, we present a proof-of-concept study in which RNA probes for in-solution hybridization capture were designed to improve the detectability of a predefined set of 12 pathogens. We validate the method by reporting enrichment rates of ∼2,000-folds for *Yersinia pestis* in six individuals from 17^th^ and 18^th^ century French plague cemeteries, enabling the detection of the plague agent with minimal sequencing. Pending further empirical validation on a broader set of microbial species, expanding our approach to target biomarkers of virtually any pathogen of interest offers a powerful tool for tracking the prevalence of infectious diseases in ancient populations.

## Highlights

• Capture probes enriching taxonomically informative markers for 12 pathogen species
• Probe set fitting the minimum capacity of commercial kits to optimize cost
• Validation on individuals from plague burials shows good enrichment performance
• Allowed *Yersinia pestis* detection in all individuals with shallow sequencing

## Graphical abstract

**Figure:**
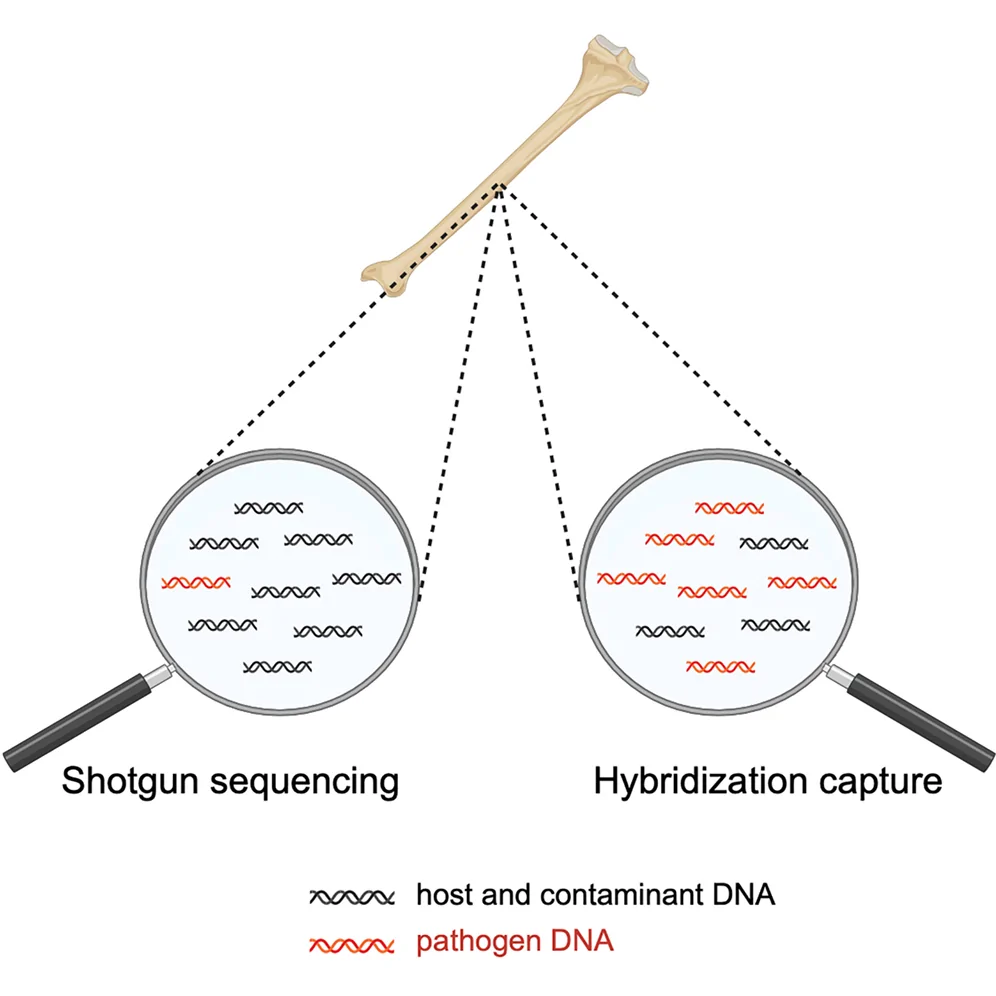

## Teaser

Microbiology; Methodology in biological sciences; Microbiological method; Archeology

## Introduction

Over the past two decades, significant advances in ancient DNA (aDNA) research have enabled the direct detection and genomic characterization of ancient pathogens whose genetic material may persist in skeletal and dental remains for thousands of years. These developments opened exciting avenues to reconstruct the epidemiological and evolutionary history of infectious agents.^1^^,^^2^

Early studies relied primarily on PCR-based assays targeting candidate pathogens responsible for historical epidemics (such as plague^3^ and smallpox^4^) or those associated with paleopathological lesions in bone (e.g., Pott disease^5^^,^^6^). The development of high-throughput sequencing, alongside improved techniques for aDNA manipulation and computational identification, has since extended our capacity to characterize the metagenomic content of ancient biological samples.^1^^,^^2^^,^^7^^,^^8^ As a result, genomic data have now been retrieved for ∼50 obligatory or opportunistic pathogens dating back ∼10,000 years,^9^ primarily from human archaeological material,^10^^,^^11^^,^^12^^,^^13^^,^^14^^,^^15^^,^^16^ although ancient pathogens have also been identified in nonhuman animals^17^^,^^18^^,^^19^^,^^20^^,^^21^^,^^22^ and plants.^23^^,^^24^ These datasets offer unprecedented insights into the past genetic composition, phylogeographic trajectories, and functional evolution of these disease agents, including ancient lineages that predate their first appearance in historical records (e.g., the “Stone Age plague,” which predates the first plague pandemic known in the historical record by thousands of years^25^^,^^26^).

Despite these advances, ancient pathogen genomics continues to face several limitations. These include the patchy availability of archaeological collections; the tissue-specificity of pathogens, limiting detection to specific infectious stages (e.g., when reaching the main bloodstream); the low prevalence of infection among sampled individuals; and differential DNA preservation in analyzed samples. Furthermore, pathogen DNA typically comprises only a tiny fraction of the total genetic material in infected remains, vastly outnumbered by host and environmental DNA, posing challenges for detection sensitivity.^8^^,^^27^^,^^28^ These constraints help explain why ancient genomic data remain sparse for most pathogens, limiting our ability to characterize past microbial genetic diversity and to make robust, quantitative inferences about past epidemiological dynamics.

While PCR amplification offers a rapid screening tool when pathogen-specific assays are available,^29^^,^^30^ it does not provide a comprehensive solution. Pathogens outside the targeted spectrum remain undetected, and the highly fragmented nature of aDNA often hinders amplification success. Moreover, issues such as nonspecific amplification, contamination, limited sequence resolution, and the inability to genetically characterize the host all constrain the utility and scope of PCR-based methods.^31^^,^^32^ In contrast, shotgun sequencing has proven effective for recovering genome-wide sequencing data from both pathogens and their hosts.^25^^,^^33^^,^^34^^,^^35^ However, this approach is resource intensive and requires high sequencing efforts and substantial postprocessing, which may be prohibitively expensive or technically demanding for many laboratories.

Hybridization capture approaches may represent more cost-effective alternatives by concentrating sequencing efforts on organisms and genomic regions of interest.^1^^,^^27^ These techniques have now been used extensively for the low-cost generation of ancient human genotyping data,^36^ as well as genome-wide data of ancient pathogens following initial detection through shallow shotgun sequencing.^16^^,^^37^^,^^38^^,^^39^ However, the systematic application of hybridization capture to increase ancient pathogen detection sensitivity has been limited so far, despite its great potential.

Early attempts at ancient pathogen detection through hybridization capture systems allowing for the parallel enrichment of multiple pathogen species have shown encouraging results—for example, the Lawrence Livermore Microbial Detection Array, or the Ancient Pathogen Screening Array.^40^^,^^41^ However, these approaches have not been used since their initial development a decade ago, calling for the design and assessment of new protocols leveraging up-to-date biomolecular technologies.

**Figure 1:**
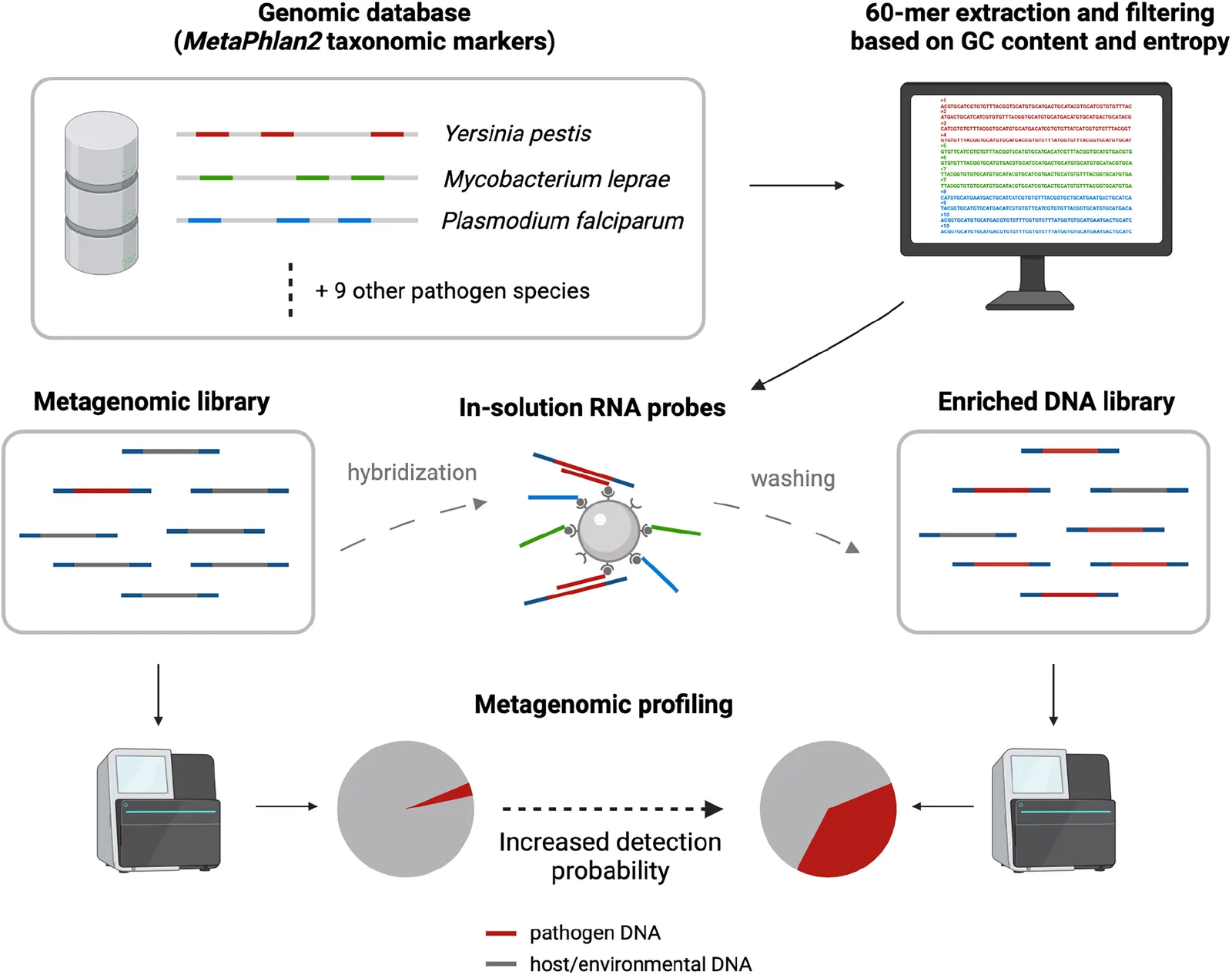
Schematic overview of the proposed hybridization capture approach aimed at the parallel detection of multiple pathogen species Taxonomically informative markers from MetaPhlAn2^42^ are used to design pathogen-specific RNA probes for hybridization capture. Ancient DNA extracts are converted into amplified, immortalized DNA libraries. During hybridization, library fragments with sequence complementarity to the RNA probes form stable, transient complexes, while nontarget DNA is washed out. This enrichment process effectively concentrates sequencing efforts on pathogen DNA potentially present in the original extract, increasing detection efficiency and reducing background noise.

Here, we present a proof-of-concept study showcasing how an in-solution hybridization capture assay targeting taxonomically informative genomic markers can theoretically enable the parallel identification of multiple human pathogen species (Figure 1). Our system employs commercially available and customizable in-solution RNA baits (myBaits; Daicel Arbor Biosciences), which have been shown to outperform capture systems based on DNA microarrays or in-solution DNA baits.^43^ While we focus our probe design on 12 selected pathogens, the assay is readily scalable to include additional species with minimal added experimental cost. The efficiency of our approach is established using skeletal remains of individuals from 17^th^- and 18^th^-century CE burials associated with documented outbreaks of bubonic plague who likely died from *Yersinia pestis* infection.^30^^,^^44^

## Results

### Capture probe set design and laboratory processing of test samples

**Figure 2:**
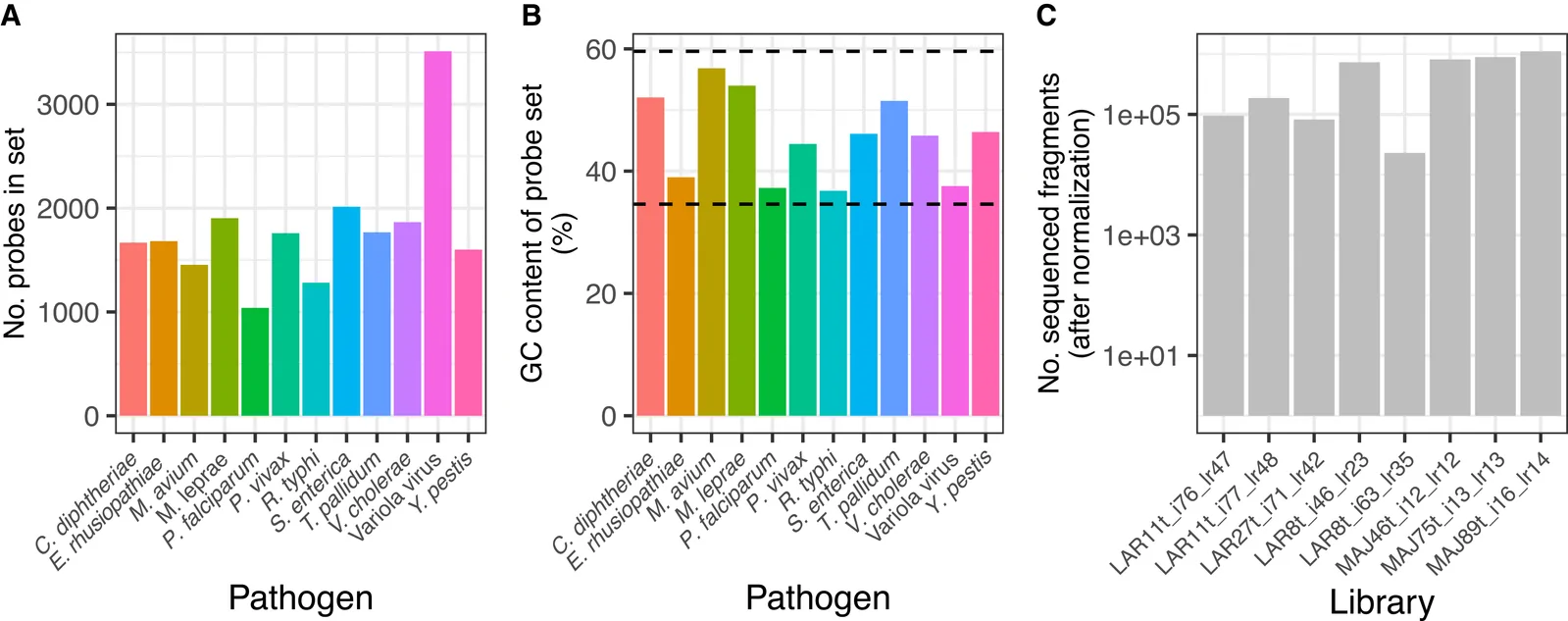
Probe set content and test sample sequencing depth (A) Number of probes included in the capture system for each target pathogen after filtering on the basis of %GC content and entropy. (B) Average %GC content of the selected probes for each pathogen. The dotted lines indicate the %GC content range recommended to ensure stable hybridization with target sequences.^45^ (C) Number of sequenced fragments for each test library after pairwise normalization of the shotgun and capture datasets.

We retrieved taxonomically informative markers (*n* = 1,036,027) from MetaPhlAn2,^42^ covering an arbitrary panel of 12 human pathogens. These were selected on the basis of their prevalence in human populations or previous aDNA literature successfully reporting their detection in ancient biological remains, and they include key candidates responsible for major historical epidemics such as plague, leprosy, syphilis, cholera, smallpox, and malaria (Figures 2A and 2B). The selected DNA sequence markers were then computationally fragmented into overlapping 60-mers with a 30-bp tiling offset. Following random downsampling and filtering on the basis of %GC content, entropy, and the absence of undetermined bases, a subset of 1,048–3,529 probes was retained per pathogen, for a total of 21,543 probes. This number was aimed at matching the minimum capacity of the myBaits Custom DNA-Seq kits, keeping experimental costs to a minimum, while allowing enrichment for a balanced set of markers for each pathogen plus the pseudocomplete smallpox genome (Figure 2A). Probe sequences are available as part of the supplemental material (Data S1).

To validate the capacity of our hybridization capture assay to enrich for pathogen DNA, we prepared eight DNA libraries from the tooth remains of six individuals: three buried between 1629 and 1630 CE at the Lariey cemetery (Alps, France; samples LAR8t, LAR11t, and LAR27t) and three buried in 1720 CE at the La Major site (Marseille, France; samples MAJ46t, MAJ75t, and MAJ89t) (Figure 2C; Table 1). Both sites are associated with major plague epidemics in France, with the former linked to outbreaks during the Thirty Years’ Wars, and the latter linked to the last historically recorded occurrence of the second pandemic in the region.^30^^,^^44^ Previous studies have identified *Y. pestis*, the etiological agent of plague, in five of these individuals through deep shotgun sequencing or targeted enrichment with a *Y. pestis*-specific capture system.^30^^,^^44^^,^^46^ All the DNA libraries were initially subjected to shallow shotgun sequencing as described in previous studies.^30^^,^^44^ These same libraries were then enriched using our hybridization capture assay, generating an additional 0.02–2.15 million read pairs per individual. We then compared the post-capture data with the initial shotgun datasets to evaluate the performance of our system. Beforehand, datasets were normalized pairwise by random downsampling to the minimum number of reads sequenced in either the shotgun or the captured library (Figure 2C).

**Table 1:** Sequencing and mapping statistics of test samples

Library	No. of reads after normalization^a^	Proportion of human fragments in shotgun	No. of *Y. pestis* reads^b^ on target (shotgun/capture)	No. of *Y. pestis* reads off-target (shotgun/capture)	Clonality after capture (on/off-target)	Fold enrichment (on/off-target)	Fold enrichment after deduplication (on/off-target)
Blanks (merged)	309,160	0	0/0	0/1	0/0	NA/NA	NA/NA
LAR11t_i76_lr47	95,153	0.043	16/33,713	891/299	0.58/0.17	2,107/0.34	891/0.28
LAR11t_i77_lr48	187,286	0.039	45/69,437	1,622/418	0.66/0.2	15,43/0.26	525/0.21
LAR27t_i71_lr42	82,391	0.067	0/140	3/0	0.38/0	>140/0	>87/0
LAR8t_i46_lr23	733,334	0.169	28/52,129	1,223/520	0.8/0.47	1,861/0.43	370/0.23
LAR8t_i63_lr35	23,015	0.168	0/1,364	42/19	0.2/0.11	>1,374/0.45	>1,092/0.4
MAJ46t_i12_lr12	818,011	0.005	1/2,246	20/14	0.97/0.57	2,246/0.7	77/0.3
MAJ75t_i13_lr13	894,010	0.006	0/945	8/8	0.96/0.5	>945/1	36/0.5
MAJ89t_i16_lr14	1,115,723	0.004	0/10	1/0	0.5/0	>10/0	>5/0

a The number of reads was equalized pairwise by random downsampling the most represented dataset to match the size of the least represented.

b High-quality alignments to the *Y. pestis* CO92 reference genome.

### *Y. pestis* DNA enrichment efficiency and impact on screening results

Only one dataset out of ten generated from experimental blanks (i.e., DNA libraries prepared from extracts performed without adding tooth sample powder) contained one single read mapping against the *Y. pestis* CO92 reference genome after hybridization capture (Table 1). In contrast, all analyzed individuals associated with plague burials yielded at least one *Y. pestis* high-quality alignment following shotgun or hybridization capture, with significant sequence variation consistent with previous results from the same samples.^30^^,^^44^ The number of high-quality alignments was sufficient to assess patterns of aDNA damage in only two of the libraries following shotgun sequencing versus four following hybridization capture^8^ (Figure S1). Evidence of postmortem deamination was limited, as reported previously for these individuals,^44^ and due to the treatment of DNA extracts with the USER enzymatic mix aimed at eliminating underlying signatures.^47^ However, base compositional profiles were marked by a substantial overrepresentation of cytosine (guanine) residues at the reference position immediately flanking read starts (ends). This confirmed successful cleavage of cytosine residues that were deaminated postmortem,^1^ thereby authenticating the generated data.

**Figure 3:**
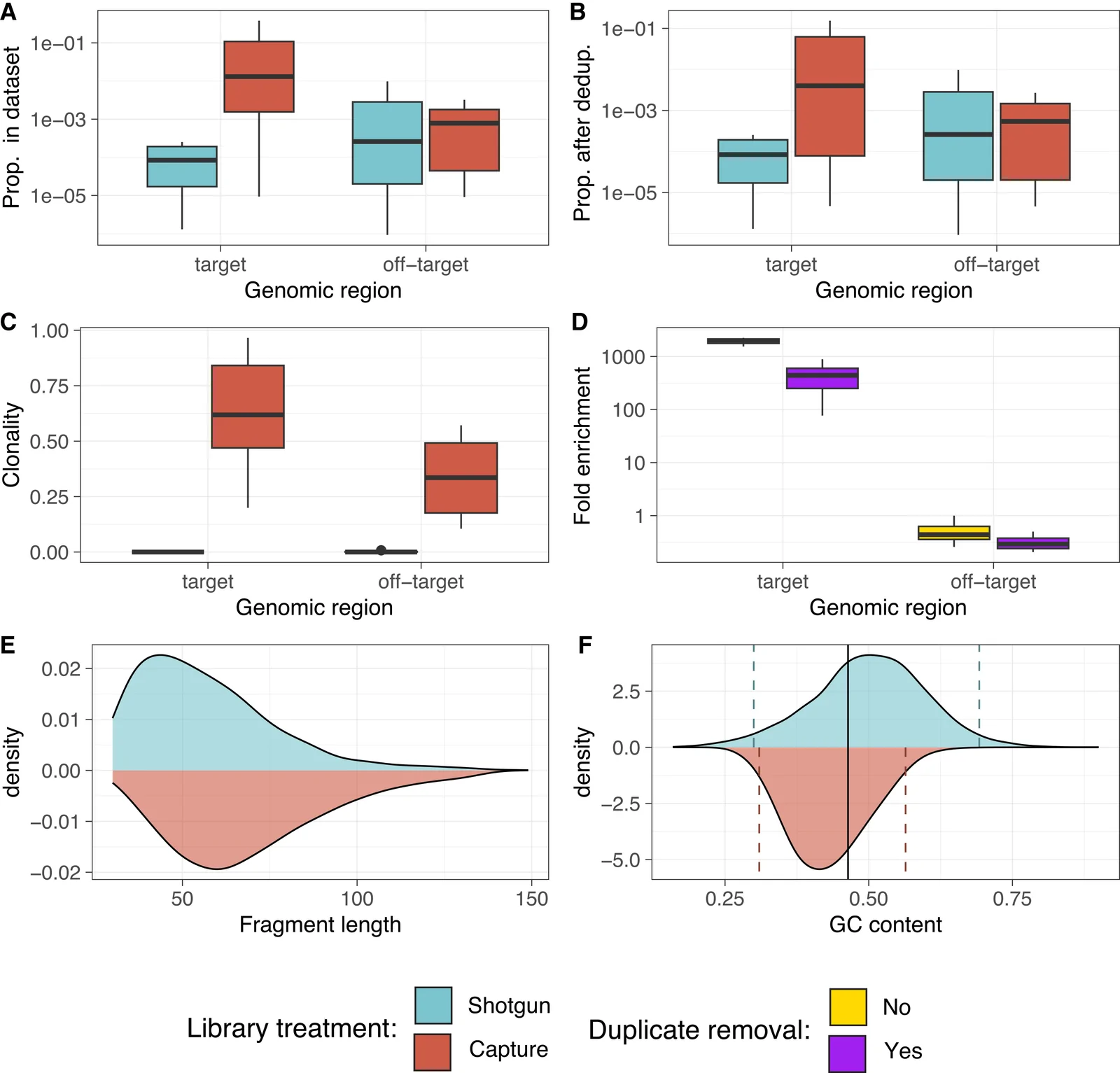
Assessment of capture efficiency (A–F) All the shotgun and capture datasets were normalized pairwise for each library and mapped against the *Y. pestis* CO92 reference genome.^48^ Different metrics were derived from high-quality *Y. pestis* alignments to assess the performance of the capture system and its impact on library content. (A) Boxplots of the fraction of *Y. pestis* fragments before and after capture on target and off-target genomic regions. (B) Boxplots of the fraction of unique (i.e., non-PCR duplicates) *Y. pestis* fragments. (C) Clonality of *Y. pestis* fragments before and after capture on target and off-target genomic regions. (D) Boxplots of *Y. pestis* DNA fold enrichment in target and off-target genomic regions. (E) Density plot showing the distribution of *Y. pestis* fragment length before and after capture. (F) Density plot showing the distribution of %GC content in *Y. pestis* fragments before and after capture. The solid black line indicates the mean %GC content of the capture probes targeting *Y. pestis.* The colored dashed lines indicate the 95% highest density intervals of the distributions.

Pairwise comparisons of shotgun and capture datasets revealed a significant increase in the number of *Y. pestis* mapping reads after capture in all cases (Table 1; paired Wilcoxon signed-rank test, *p* value = 0.004). This corresponds to an average enrichment of 54-fold (range = 10- to 119-fold). The enrichment was especially notable in the genomic regions targeted by the capture probes, reaching an average of 1,939-fold (range = 1,543- to 2,246-fold; Figures 3A and 3B). By contrast, non-target genomic regions appeared depleted after capture (average fold enrichment: 0.4; Figures 3A and 3B). The clonality of *Y. pestis* alignments significantly increased following hybridization capture (Figure 3C; paired Wilcoxon signed-rank test, *p* value = 0.004), with an average of 63% (20%–97%) on target genomic regions, indicating that capture allowed recovery of most of the targeted genomic material contained in the libraries even with shallow sequencing efforts. Notably, the clonality of *Y. pestis* alignments falling outside of target regions also increased (paired Wilcoxon signed-rank test, *p* value = 0.016). This may reflect a combination of enrichment outside of targeted regions due to daisy chaining,^44^^,^^45^ and the loss of library complexity due to the additional amplification cycles performed to obtain sufficient material for capture. The latter effect is difficult to precisely quantify in our case due to the small number of *Y. pestis* fragments recovered prior to capture, precluding estimates of library complexity in shotgun datasets. The potential resulting loss of library complexity should, however, not represent a major issue for our approach, which is solely aimed at facilitating detection by enriching short diagnostic genomic regions rather than sequencing whole genome sequences.

Enrichment was significantly biased toward longer DNA fragments (Kolmogorov-Smirnov test, *p* values < 1e‒15), with median lengths of high-quality alignments equalling 53 and 66 bp before and after hybridization capture, respectively (Figure 3E), in line with previous work on hybridization capture.^44^^,^^49^ This size shift likely results from the improved stability of probe-template dimers when templates can anneal to the whole probe RNA strand. Similarly, the base composition (%GC content) of high-quality alignments after hybridization capture reflects that of the probe set, as expected following successful enrichment. It was indeed significantly impacted following hybridization capture (Kolmogorov-Smirnov test, *p* values < 1e‒15) and showed a shrunken distribution around the base composition of the probe set.

The analyses above measured the capture efficiency through raw comparisons of high-quality alignments against the *Y. pestis* genome, all of which are expected to hybridize preferentially to the capture probes regardless of their actual taxonomic origin. However, highly similar results were obtained when restricting the analyses to DNA fragments identified as *Y. pestis* through metagenomic profiling (Figure S2).

**Figure 4:**
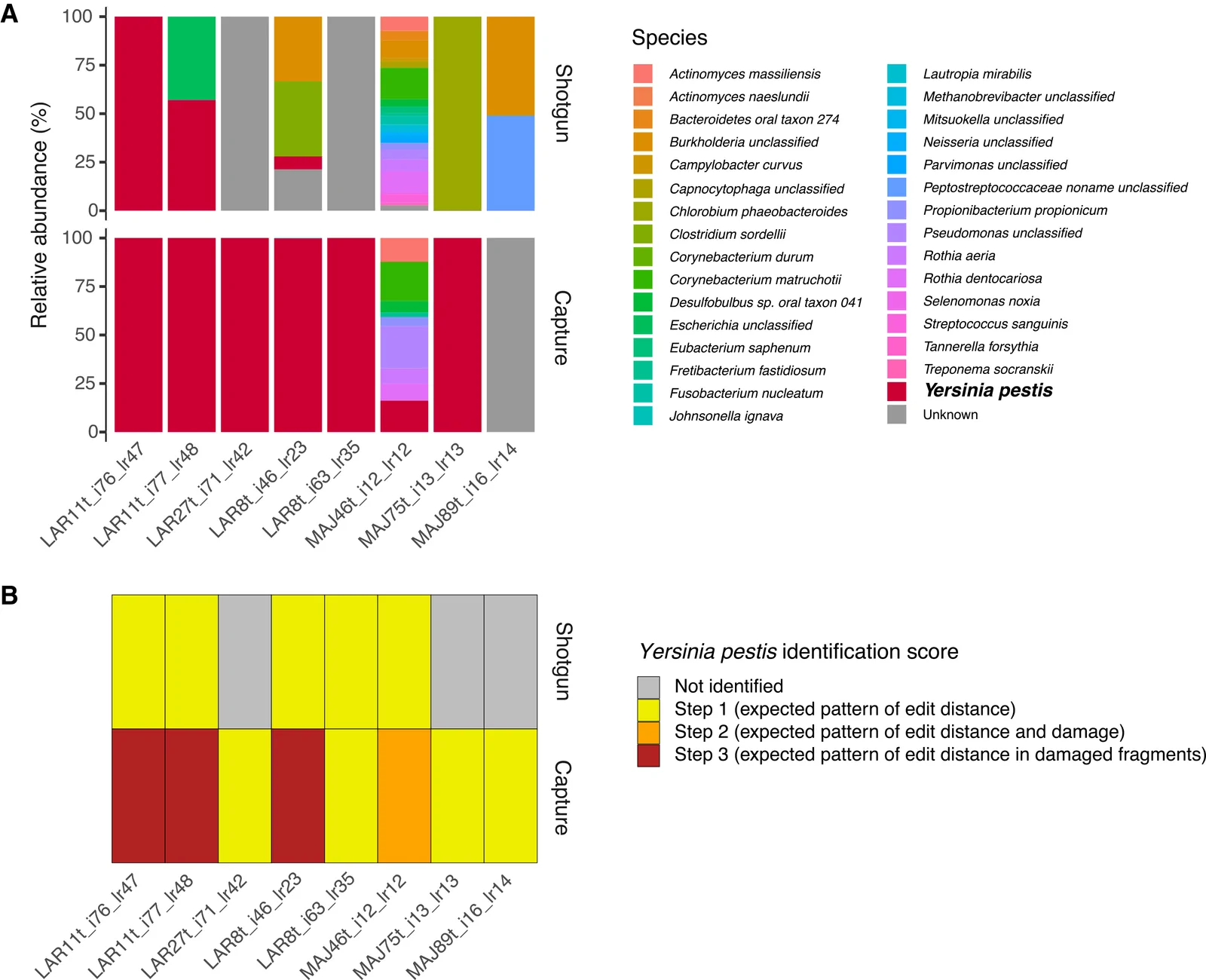
Metagenomic profiling and pathogen screening (A) Results of metagenomic profiling with the metaBIT pipeline.^50^*Y. pestis* becomes the dominant bacterial species in most libraries following hybridization capture. (B) Results of *Y. pestis* detection using the HOPS ancient pathogen screening pipeline.^51^ None of the samples yielded specific hits to the other pathogen species represented in our probe set.

To evaluate whether our system could allow the detection of *Y. pestis* using more specific assignment methods, we first performed metagenomic profiling of the datasets using the metaBIT pipeline,^50^ relaxing the minimum default relative abundance threshold (from 1% to 0%). *Y. pestis* was detected in three out of eight of the shotgun datasets (Figure 4A). In contrast, it represented the dominant bacterial species in all the libraries after capture, with the exception of MAJ89t_i16_lr14, for which no species could be detected. Importantly, none of the other species targeted by our probe set was detected in any of the samples after capture. Combined, these analyses provide confidence regarding the specificity and sensitivity of our approach, although we acknowledge that more representative archaeological negative controls could have been used in addition to laboratory blanks to evaluate specificity with respect to *Y. pestis* detection.

To further assess the authenticity of the *Y. pestis* findings, we screened our datasets using the HOPS ancient pathogen pipeline,^51^ building an index containing one representative of all bacterial species included in the NCBI RefSeq database. These analyses confirmed the presence of *Y. pestis* in 5 out of 8 shotgun-sequenced libraries, with a minimum identification step of 1 (indicating expected patterns of edit distance; Figure 4). After capture, all eight libraries were positive for *Y. pestis*, and four of them presented identification scores >1, indicating signatures of aDNA damage in assigned reads, despite minimal deamination patterns due to USER treatment (Figure S1). Applying HOPS to the full dataset instead of *Y. pestis* alignments only (a strategy used to reduce computational burden) provided similar results (Figure S3). In particular, this did not yield any hits for any other pathogen species, further supporting the specificity of our approach.

## Discussion

Identifying the etiological agents responsible for past epidemics remains challenging in the absence of genetic evidence confirming the presence of specific pathogens. Postmortem molecular degradation significantly limits aDNA preservation and recovery, reducing the power of genetic identification. Additionally, many pathogens exhibit specific tropism for soft tissues—which are rarely preserved—making detection impossible, unless pathogens penetrate the overall blood circulation or form lesions in the calcified skeletal and dental remains typically recovered during archaeological excavations.

Although highly specific, PCR-based detection assays often yield a high rate of false negatives when applied to highly fragmented DNA typical of ancient samples.^52^ Such assays also consume a considerable portion of the aDNA extract per detection attempt, while returning only limited sequence information, which represents a serious issue when extraction is destructive and sample material is scarce. In contrast, shotgun sequencing is perhaps the most comprehensive and conservative approach, offering a relatively unbiased snapshot of all DNA templates present in a sample. However, even this method is subject to biases introduced during library preparation and amplification, which can distort base composition and fragment size distribution depending on the underlying Taq DNA polymerases used.^53^ More critically, shotgun sequencing is resource intensive, as pathogen DNA often represents only a minute fraction of the total metagenomic content, requiring deep sequencing to detect target organisms reliably.

Hybridization capture provides a practical compromise between these two approaches. It combines the specificity of targeted detection with reduced sequencing demands, making it a cost-effective and efficient alternative. In this study, we established that a simple hybridization capture assay can significantly improve the detection of pathogens, confirming the presence of *Y. pestis* in all six ancient individuals tested. In contrast, shotgun sequencing of the same samples identified only two (metaBIT; Figure 4A) or three (HOPS; Figure 4B) individuals positive for this infection. Excluding sequencing costs, the experimental hybridization procedure used in this study is associated with an estimated per-sample cost of approximately 100–170 euros, a figure that might vary across laboratories and can be further reduced by multiplexing a greater number of double-indexed DNA libraries (up to 20+) during co-capture, as previously demonstrated.^44^^,^^49^ Assuming a minimal sequencing cost of €1/M sequencing reads, a maximum cost of €170 for capture, and an enrichment efficiency of ∼2,000-fold (the average value observed in our tests), we estimate that capture should be largely cost effective for the retrieval of target pathogen DNA under most scenarios (Figure S4). However, we acknowledge that this estimate does not account for the additional laboratory work required and that the cost efficiency of the approach depends on the optimal sequencing effort that can vary across study aims and samples.

Hybridization capture is also inherently scalable; the range of targeted pathogens can be expanded by increasing probe diversity, potentially encompassing the full spectrum of DNA viruses, bacteria, fungi, and parasites commonly associated with human infections. Among perspectives for future improvement, the methodology proposed could be extended to target genomic positions permitting direct lineage assignment of ancient pathogen strains. Co-enriching genome-wide data from the host, including from the immunogenome, might also offer an opportunity to track co-evolutionary arm races between pathogens and their hosts, although the impact of imbalanced host and pathogen DNA proportions on capture efficiency should be assessed. Provided that sufficient sequence data are available for designing probes, our approach can also be extended to nonhuman hosts to explore pathogen loads across broader ecological and evolutionary contexts. Such designs might be used on environmental samples, which represent promising alternative sources of ancient pathogen DNA, particularly for gastrointestinal germs, which are difficult to detect from skeletal remains.^54^^,^^55^ Finally, our approach is poised for the identification of co-infection cases caused by multiple pathogen species. Compared with shotgun sequencing, the increased sensitivity of hybridization capture is likely to yield more accurate estimates of pathogen prevalence in ancient populations. This could, in turn, offer a critical step toward the reconstruction of past health landscapes and the understanding of how susceptibility to infectious diseases has varied historically in response to environmental, sanitary, and social conditions.

In the future, additional experiments could be conducted to further optimize the cost-performance trade-off of this approach. While our results suggest that the minimal assay presented here was sufficient to recover most of the targeted *Y. pestis* genomic material in our test samples, increasing probe numbers might improve detection for other pathogen species or more challenging study material. Reciprocally, one could assess whether decreasing the number of probes per pathogen species could allow broadening the target range without compromising performance or inflating experimental costs. In this study, we used *MetaPhlAn2* reference database as a basis for capture probe set design to match the metagenomic profiling pipeline implemented in our laboratory, thereby ensuring workflow consistency. The use of more updated reference databases could further improve the approach by providing more accurate taxonomic assignments and extended genetic diversity for probe design, limiting potential enrichment deficiencies due to reference biases. Nevertheless, our results did not suggest any major issue in this regard, and previous studies have demonstrated successful genome-wide enrichment of ancient pathogen strains using capture probes designed from modern references that might diverge significantly from the target.^26^^,^^39^ To conclude, while our approach can be readily used with good expected performance, it also provides multiple avenues for future improvement and broader application.

### Limitations of the study

This study has started to unlock the specificity and sensitivity of in-solution hybridization capture assays to detect a range of human pathogen species on ancient osseous remains. However, the system could be experimentally validated only for *Y. pestis*, as this was the only pathogen for which material with a confident diagnosis of infection was readily available in our laboratory at the time of this study. Because the RNA probes for all pathogens were designed using the same standardized method, we can reasonably expect comparable performance for the additional targets included in the assay. Nevertheless, empirical validation on a more diverse set of species is needed to confirm the capacity of the approach to co-detect multiple infections or any of the eleven other pathogens targeted. Experimental validation was also limited to relatively recent ancient specimens from only two preservation contexts. The broader performance on a more comprehensive set of samples, comprising the full range of DNA preservation levels and chronological depths represented in the archaeological record, remains to be established. Finally, the limited sample size tested prevents a comprehensive statistical assessment of performance, and caution is warranted in the interpretation of the highly significant *p* values presented.

## Resource availability

### Lead contact

Requests for further information and resources should be directed to and will be fulfilled by the lead contact, Arthur Kocher (arthur.kocher@gmail.com).

### Materials availability

This study did not generate new materials.

### Data and code availability

• All sequencing data generated in the context of this study can be found in the European Nucleotide Archive (ENA: PRJEB89986). Probe sequences are provided as the supplemental information.
• This article does not report original code.
• Any additional information required to reanalyze the data reported in this paper is available from the lead contact upon request.

## Acknowledgments

This research was funded by the CNRS MITI (Mission pour les Initiatives Transverses et Interdisciplinaires) IndigenousHealth program, the ANR LifeChange, the Simone and Cino Del Duca Foundation (Subventions scientifiques 2020, HealthTimeTravel), and the French government under the France 2030 investment plan, as part of the Initiative d’Excellence d’Aix-Marseille Université (AMIDEX AMX—22-RE-AB-055). A.K. and A.S.-O. were supported by the European Union’s Horizon Research and Innovation Program (MSCA grant agreement 101153422-VIRODOM and ERC-StG grant agreement 101117101-anthropYXX, respectively). We thank Dr. Catherine Thèves, Dr. Clio Der Sarkissian, and Pr. Norbert Telmon for their contributions to the original paleogenomic studies involving some of the individuals analyzed here, Laure Calvière-Tonasso and Stéphanie Schiavinato for the CAGT ancient DNA facilities maintenance, as well as all the members of the AGES group at CAGT for fruitful discussion, and Antoine Lacombe for hardware maintenance of the server cluster.

## Author contributions

L.O. designed the study; S.T., M.S., C.C., and L.O. provided the study material; A.S.-O. and P.C. performed the experimental work; A.K. and G.L. performed the bioinformatic analysis; A.K., A.S.-O., and L.O. wrote the manuscript, with input from all co-authors.

## Declaration of interests

The authors declare no competing interests.

## STAR★Methods

### Key resources table

**Table:** 

REAGENT or RESOURCE	SOURCE	IDENTIFIER
**Biological samples**		

Osteological remain	Seguin-Orlando et al.^30^	LAR8
Osteological remain	Seguin-Orlando et al.^30^	LAR11
Osteological remain	Seguin-Orlando et al.^30^	LAR27
Osteological remain	Clavel et al.^44^	MAJ45
Osteological remain	Clavel et al.^44^	MAJ75
Osteological remain	Clavel et al.^44^	MAJ89
Ancient DNA library	Seguin-Orlando et al.^30^	LAR8t_i63_lr35
Ancient DNA library	Clavel et al.^44^	LAR8t_i46_lr23
Ancient DNA library	Seguin-Orlando et al.^30^	LAR11t_i76_lr47
Ancient DNA library	Seguin-Orlando et al.^30^	LAR11t_i77_lr48
Ancient DNA library	Seguin-Orlando et al.^30^	LAR27t_i71_lr42
Ancient DNA library	Clavel et al.^44^	MAJ46t_i12_lr12
Ancient DNA library	Clavel et al.^44^	MAJ75t_i13_lr13
Ancient DNA library	Clavel et al.^44^	MAJ89t_i16_lr14

**Chemicals, peptides, and recombinant proteins**		

N-Lauroylsarcosine solution 30% 500 mL	Dutscher	Cat#348533
Proteinase K 10 MG	Thermo Fisher Scientific	Cat#AM2542
H2O, Molecular Biology Grade, Fisher BioReagents	Thermo Fisher Scientific	Cat#10977015
Tween 20 100 ML	Thermo Fisher Scientific	Cat#13464259
Ethanol, Absolute, Mol Biology Grade	Thermo Fisher Scientific	Cat#16606002
USER Enzyme	New England Biolabs	Cat#M5505L
NEBNext End Repair Module	New England Biolabs	Cat#E6050L
Bst DNA POLYMERASE	New England Biolabs	Cat#M0275L
NEBNext Quick Ligation Module	New England Biolabs	Cat#E6056L
BSA Molecular Biology Grade	New England Biolabs	Cat#B9000S
Accuprime PFX DNA Polymerase 100 ML	Thermo Fisher Scientific	Cat#10472482
Agencourt AMPure XP - 60 mL	Thermo Fisher Scientific	Cat#A63881
Buffer PE	QIAGEN	Cat#19065
Buffer PB	QIAGEN	Cat#19066
Buffer EB	QIAGEN	Cat#19086
dNTP Set 100 mM 100 mL	Thermo Fisher Scientific	Cat#10297018
EDTA 0.5M pH 8.0 Fisher Bioreagents 500 ML	Thermo Fisher Scientific	Cat#AM9261

**Critical commercial assays**		

MinElute PCR Purification Kit	QIAGEN	Cat#28006
Tapestation screenTape D1000 HS	Agilent	Cat#5067-5584
Qubit dsDNA HS Assay Kit	Thermo Fisher	Scientific Cat#Q32854
MiniSeq High Output Reagent Kit (150-cycles)	Illumina	Cat#FC-420-1002
myBaits Custom DNA-Seq	Arbor Biosciences	Cat#300596.v5

**Deposited data**		

Sequencing dataset	This study	ENA: PRJEB89986

**Software and algorithms**		

AdapterRemoval2 v. 2.3.0	Schubert et al.^56^	https://github.com/MikkelSchubert/adapterremoval
SeqKit v. 2.4.0	Shen et al.^57^	https://github.com/shenwei356/seqkit
fastp v. 0.23.2	Chen et al.^58^	https://github.com/opengene/fastp
Bowtie 2 v. 2.5	Langmead and Salzberg^59^	https://github.com/BenLangmead/bowtie2
SAMtools v. 1.17	Li and Durbin^60^	https://github.com/samtools/samtools
Picard Tools v. 2.18.7	Broad Institute	http://broadinstitute.github.io/picard/
mapDamage2 v. 2.0.9–2	Jónsson et al.^61^	https://ginolhac.github.io/mapDamage
BEDTools v. 2.31.1	Quinlan and Hall^62^	https://github.com/arq5x/bedtools2
metaBIT v. 1.0.1	Louvel et al.^50^	https://bitbucket.org/Glouvel/metabit/src/master/
HOPS v. 0.35	Hübler et al.^51^	https://github.com/rhuebler/HOPS

### Method details

#### Probe set design for the parallel enrichment of 12 human pathogens

We designed a custom *myBaits* hybridization capture kit (Daicel Arbor Biosciences) to enrich taxonomically informative genomic regions in 12 human pathogens representing a broad spectrum of common infectious diseases: *Corynebacterium diphtheriae* (the agent of diphtheria), *Erysipelothrix rhusiopathiae* (erysipeloid), *Mycobacterium avium* (nontuberculous mycobacteriosis), *Mycobacterium leprae* (leprosy), *Plasmodium falciparum* and *Plasmodium vivax* (malaria), *Rickettsia* sp. (typhus), *Salmonella enterica* (typhoid fever and other salmonelloses), *Treponema pallidum* (syphilis, yaws and bejel), variola virus (smallpox), *Vibrio cholerae* (cholera), and *Yersinia pestis* (bubonic plague). This arbitrary set of pathogens was selected on the basis of their prevalence in human populations or previous ancient DNA literature successfully reporting their detection in ancient human,^10^^,^^13^^,^^34^^,^^37^^,^^44^^,^^63^^,^^64^ and nonhuman material (for *E. rhusiopathiae*).^22^^,^^65^ For each of these pathogens, with the exception of variola virus, we designed hybridization probes targeting the genomic regions included in the *MetaPhlAn2* reference database (v20_m200), which consists of clade-specific marker genes allowing unambiguous taxonomic assignments.^42^ 60-mers were extracted from each marker gene via 30-bp tiling. For variola virus, 60-mers were extracted from the full reference genome (GenBank: NC_001611.1). Only probes containing 34.6%–59.6% GC and showing entropy values ≥0.5427 were retained to ensure stable hybridization with target sequences and sufficient sequence complexity,^45^ leaving 1,282–18,767 candidate probes per pathogen. All of the 1,282 and 1,903 candidate probes were selected for *Rickettsia* sp. (typhus) and *Mycobacterium leprae* (leprosy), respectively. For each remaining pathogen, unique probe sequences were then randomly sampled down to 2,140, except for variola virus, for which all 3,529 60-mers could be retained while remaining within the limit of ∼20,000 probes corresponding to the minimum capacity of the myBaits Custom DNA-Seq kits. Finally, only unique probes were kept, leaving a total of 21,543 unique probes for synthesis. This procedure was followed to optimize experimental costs pertaining to probe synthesis, while potentially allowing for whole-genome enrichment of variola virus DNA. Limiting the number of variola virus probes within the range targeted for other species would have instead allowed for the detection of an additional pathogen without any additional experimental cost.

#### Laboratory protocol and test samples

The efficiency of our capture system was evaluated on aDNA libraries prepared from the dental remains of six individuals excavated from graves associated with historic epidemics of bubonic plague in France. These included three individuals from the 1629–1630 CE plague cemetery of Lariey-Puy-Saint-Pierre in the French Alps (LAR) and three individuals from the site of La Major (MAJ) associated with the Great Plague of Marseille of 1720–1722CE^30^^,^^44^ (Method S1). Given the burial contexts, all of these individuals were likely to have died from an infection with *Y. pestis.* This was previously confirmed for five of them via polymerase chain reaction (PCR) of a 133-bp fragment located on the *pla* gene (LAR8, LAR11 and LAR27^30^), shotgun sequencing data (LAR8 and LAR11^30^), or following enrichment using a *Y. pestis* whole-genome capture system (LAR27, MAJ46 and MAJ75^44^).

Molecular work, from dental pulp sampling to sequencing library building and PCR setup preparation, was carried out in the ancient DNA facilities of the CAGT (Université de Toulouse, France), following strict procedures to avoid and monitor contamination risks. Illumina sequencing libraries LAR11t_i76_lr47, LAR11t_i77_lr48, LAR8t_i63_lr35 and LAR27t_i71_lr42 were prepared for the study from Seguin-Orlando and colleagues,^30^ and libraries LAR8t_i46_lr23, MAJ46t_i12_lr12, MAJ75t_i13_lr13 and MAJ89t_i16_lr14 were prepared for the study from Clavel and colleagues.^44^ Briefly, 40–70 mg of dental pulp was powdered as described by Neumann and colleagues.^66^ The DNA extraction was performed following a modified version of the Y2 protocol described by Gamba and colleagues,^67^ with a predigestion step of 1 h at 37 °C, followed by full digestion of the remaining pellet overnight at 42 °C. An aliquot of 200 μL of the overnight digestion lysate was purified on a MinElute column (QIAGEN©) and subjected to Uracil-Specific Excision Reagent (USER, NEB) treatment. The sequencing libraries were constructed following a protocol described by Rohland et al.^47^ and modified as described by Fages et al.^68^ to introduce a unique 7-nucleotide barcode within both adapters P5 and P7 (identified with the *_lrXX* suffix appended to the library name).

Libraries for shotgun sequencing were PCR enriched and indexed by performing 10–12 PCR cycles in a 25 μL total reaction volume, using 1 unit of AccuPrime™ Pfx DNA polymerase, 4 μL of unamplified DNA library, the InPE1.0 primer and one custom PCR primer (including a unique 6-nucleotide index identified as the *_iXX* suffix in the library name, both at a final concentration of 200 nM). The amplified products were purified using Agencourt Ampure XP beads (1.4:1 bead:DNA ratio) and eluted in 20 μL of EB+0.05% tween.

Before capture, libraries were PCR enriched and indexed as described previously^44^ by performing two consecutive rounds of PCR amplification. For the first amplification round, 8 parallel amplifications of 8 cycles were performed in a volume of 25 μL, using 2 μL of the unamplified DNA library, 1 unit of AccuPrimeTM Pfx DNA polymerase, the InPE1.0 primer and one custom-indexed PCR primer (200 nM final concentration). The products were pooled two by two, purified on four parallel MinElute columns (QIAGEN©), and eluted in 11 μL of EB+0.05% tween each. Each of the four purified amplification products was split and processed through two parallel PCR amplifications for 10 cycles. For each PCR, 1 μL of the eluate was added to a 25 μL total reaction volume. This second round of PCR was carried out under the same conditions as those used for the first round of amplification, except that IS5 and IS6 were used as PCR primers.^69^ The resulting eight PCR products for each library were copurified on a single MinElute column (QIAGEN©) and eluted in 12 μL of EB+0.05% tween. Post-PCR library size profiles were checked on a Tapestation 4200 instrument (Agilent Technologies), and concentrations were measured on a QuBit HS dsDNA assay (Invitrogen). Before capture, the three MAJ libraries were pooled together to obtain a final DNA quantity of 2.546 mg (0.412 mg of MAJ46t_i12_lr12, 1.052 mg of MAJ75t_i13_lr13 and 1.082 mg of MAJ89t_i16_lr14), whereas the five LAR libraries pooled together total 2.489 mg of DNA (0.548 mg for LAR11t_i76_lr47, 0.968 mg for LAR11t_i77_lr48, 0.031 mg for LAR8t_i63_lr35, 0.844 for LAR8t_i46_lr23 and 0.098 mg for LAR27t_i71_lr42). Pools were concentrated on a MinElute column to a final volume of 7 μL. Capture was performed according to the *myBaits* High Sensitivity Protocol Version 5.00, with two rounds of 18–22 h of hybridization with the probes at 55 °C. The captured libraries were PCR amplified for 14 cycles between both rounds and for 8 cycles after the second capture round (both using the IS5 and IS6 primers and similar conditions as those used for the precapture PCRs).

Each final library, or library pool, was checked for size, molarity and concentration on a Tapestation 4200 instrument (Agilent Technologies) and on a QuBit HS dsDNA assay (Invitrogen) before being sequenced in paired-end mode on an Illumina MiniSeq instrument (2x81 cycles) at CAGT (Université de Toulouse, France) or on a NovaSeq 6000 (2x150 bp reads) at SciLifeLab (Stockholm, Sweden).

### Quantification and statistical analysis

Sequencing read demultiplexing and collapsing, as well as adapter and poor-quality end trimming, were performed using *AdapterRemoval2* v. 2.3.0,^56^ tolerating at most one mismatch in each internal barcode (–barcode-mm 1 –minadapteroverlap 3 –mm 5). Resulting merged and unmerged reads were concatenated for each dataset. The number of reads for the shotgun and capture datasets were then equalized pairwise for each library by random downsampling using *SeqKit* v. 2.4.0^57^ to provide fair comparisons in subsequent analyses. To limit unspecific mapping to the *Y. pestis* genome, reads exhibiting low quality, low complexity or a length shorter than 30 bp were then removed using *fastp* v. 0.23.2^58^ (using --length_required 30 --n_base_limit 3 --low_complexity_filter --trim_poly_g and default parameters otherwise).

All preprocessed datasets were then mapped against the human T2T-CHM13v2.0 reference genome^70^ using *Bowtie 2* v. 2.5^59^ (with the --fast parameter setup) to compute the percentage of human fragments in each dataset, which were then filtered out for subsequent analyses. The remaining reads were mapped against the *Y. pestis* CO92 reference genome^48^ using *Bowtie 2* v. 2.5 (with the --sensitive parameter setup), and alignments with a minimum mapping quality of 20 were extracted (*samtools view -c -F 4 -q 20*). PCR duplicates were removed using *MarkDuplicates* from Picard Tools (version 2.18.7, http://broadinstitute.github.io/picard/). Damage patterns and base composition profiles of *Y. pestis* high-quality alignment (minimum mapping quality of 20) were computed using mapDamage2,^61^ and considering base with quality scores greater than or equal to 30. Capture probe sequences were mapped against the *Y. pestis* CO92 reference genome following the same procedure to extract the genomic coordinates of the targeted regions using the *BEDTools* v. 2.31.1^62^ (*bamtobed*, *sort* and *merge* commands).

To measure the enrichment efficiency of the hybridization capture system, we computed the ratio of the number of *Y. pestis-*mapping reads between the pairwise capture and shotgun datasets. This enrichment factor was computed considering all genome positions globally, as well as for on-target and off-target regions separately (using the *samtools view -L* option with the *bed* file generated from the probe alignment as aforementioned). To assess the presence of an expected enrichment bias toward long and GC-rich DNA molecules, we further compared the fragment size and %GC content distribution of *Y. pestis*-mapping fragments in capture and shotgun datasets. Only merged reads with a length <150 bp were considered for these comparisons, approximately corresponding to the maximum fragment length achievable with 2x81 cycles given the parameters considered when using *AdapterRemoval2*.^56^ The statistical significance of the observed differences was assessed using paired Wilcoxon signed-rank tests and Kolmogorov-Smirnov tests. To assess the impact of unspecific hybridization on these measurements, these analyses were repeated focusing on the strict set of sequences identified as *Y. pestis* by the *HOPS* pipeline (see below).

To assess the presence of *Y. pestis* DNA in the libraries more accurately, metagenomic profiling was performed using the *metaBIT* pipeline,^50^ restricting assignments to nonviral and noneukaryotic taxa present in the *MetaPhlAn2* database (v20_m200).^42^ Furthermore, we screened our datasets using the *HOPS* ancient pathogen pipeline,^51^ with an index containing one representative of all bacterial species included in the NCBI RefSeq database (with *Malt* parameters: *--minPercentIdentity 85, --topPercent 1, --gapOpen 1000, --maxAlignmentsPerQuery 100, --maxAlignmentsPerRef 10, --minSupport 1*). Those reads mapping against the *Y. pestis* CO92 reference genome were initially used as input for HOPS to reduce computational costs. Then, the analysis was repeated considering the full datasets (following filtering of human alignments) to further assess the specificity of the approach.

## Supplemental information

Document S1. Figures S1–S4 and Method S1

Data S1. Hybridization capture probe set in fasta format
