# Supplementary material for: A minimal hybridization capture approach for the parallel enrichment and cost-effective detection of ancient human pathogens
Source: iScience. 2026 Aug 20;29(9):116625. doi: 10.1016/j.isci.2026.116625 (PMC13594432; doi:10.1016/j.isci.2026.116625)
Supplement: Supplementary file 1 [file mmc1.pdf]

iScience, Volume *29*

Supplemental information

A minimal hybridization capture approach for the parallel enrichment and cost-effective detection of ancient human pathogens

Arthur Kocher, Andaine Seguin-Orlando, Pierre Clavel, Guillaume Louvel, Richard Jonvel, Stéfan Tzortzis, Michel Signoli, Caroline Costedoat, and Ludovic Orlando

Supplemental figures

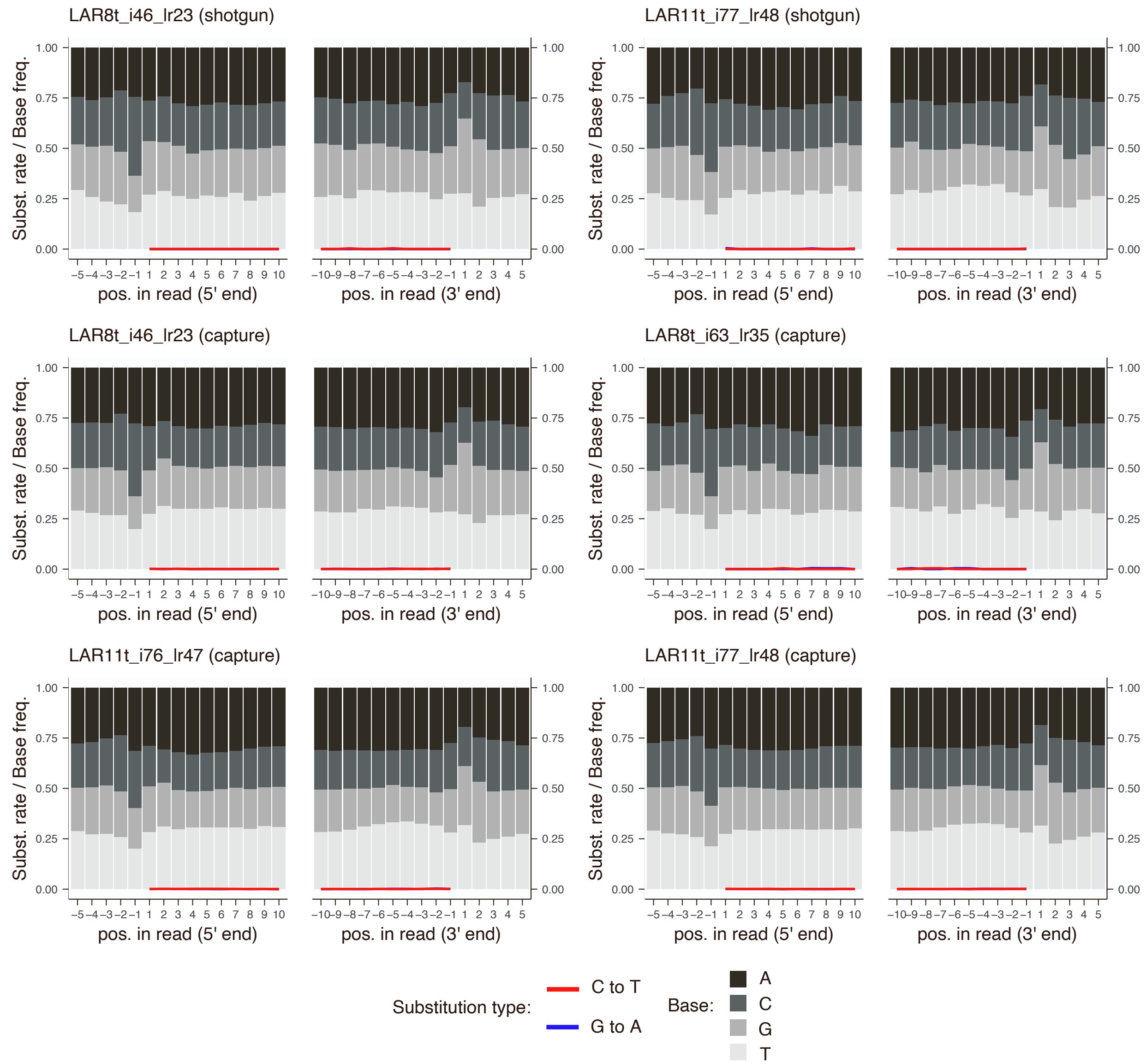

Figure S1: Damage patterns and base composition profiles of *Y. pestis* high-quality alignments following hybridization capture, for the four libraries yielding more than 1,000 unique alignments [S1]**.** All aligned reads were processed using mapDamage2 [S2], considering base with quality scores superior or equal to 30. Position-wise base composition profiles are provided for the 10 first (left, 1 to 10) and 10 last (right, -10 to -1) read positions within sequencing reads, and the 5 positions preceding read starts (left, -5 to - 1) or following read ends (right, 1 to 5) on the reference genome. As the libraries were USER-treated, the DNA damage signature is tracked through an increase in %C and %G at the alignment positions immediately preceding and following read termini, respectively [S3].

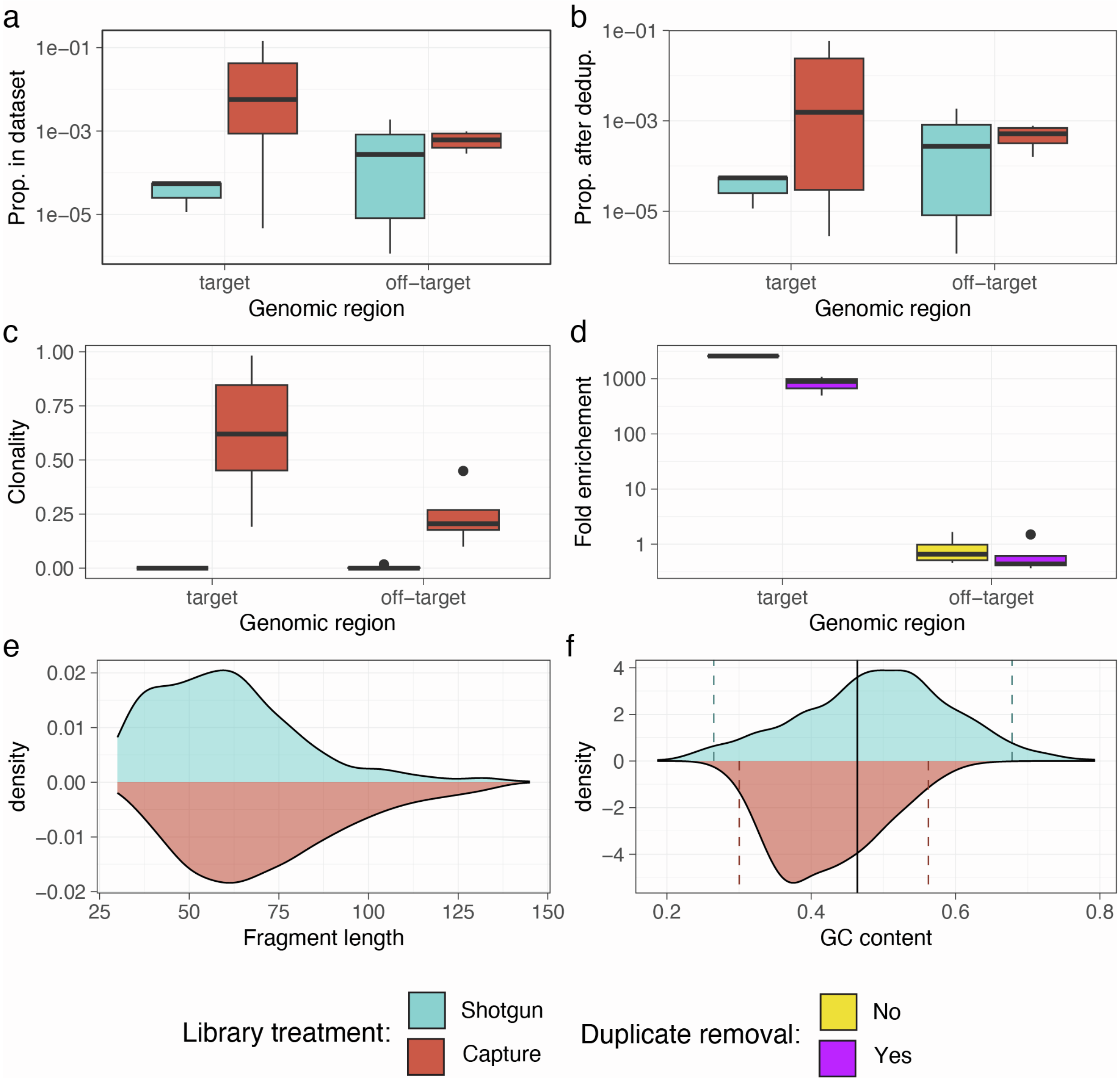

Figure S2: Assessment of capture efficiency using *Yersinia pestis*-specific reads. Shotgun and capture datasets were normalized pairwise for each library to represent identical sequencing efforts before being used as input for metagenomic profiling with the *HOPS* ancient pathogen screening pipeline[4]. Reads classified as *Y. pestis* were then mapped against the *Y. pestis* CO92 reference genome. Different metrics were derived from high-quality *Y. pestis* alignments to assess the performance of the capture system and its impact on library content. **(a)** Boxplots of the fraction of *Y. pestis* fragments before and after capture in target and off-target genomic regions. **(b)** Boxplots of the fraction of unique (*i.e.* non-PCR duplicates) *Y. pestis* fragments. **(c)** Clonality of *Y. pestis* fragments before and after capture in target and off-target genomic regions. **(d)** Boxplots of *Y. pestis* DNA fold enrichment in target and off-target genomic regions. **(e)** Density plot showing the distribution of *Y. pestis* fragment length before and after capture. **(f)** Density plot showing the distribution of %GC content in *Y. pestis* fragments before and after capture. The solid black line indicates the mean %GC content of the capture probes targeting *Y. pestis.* The colored dashed lines indicate the 95% highest density intervals of the distributions.

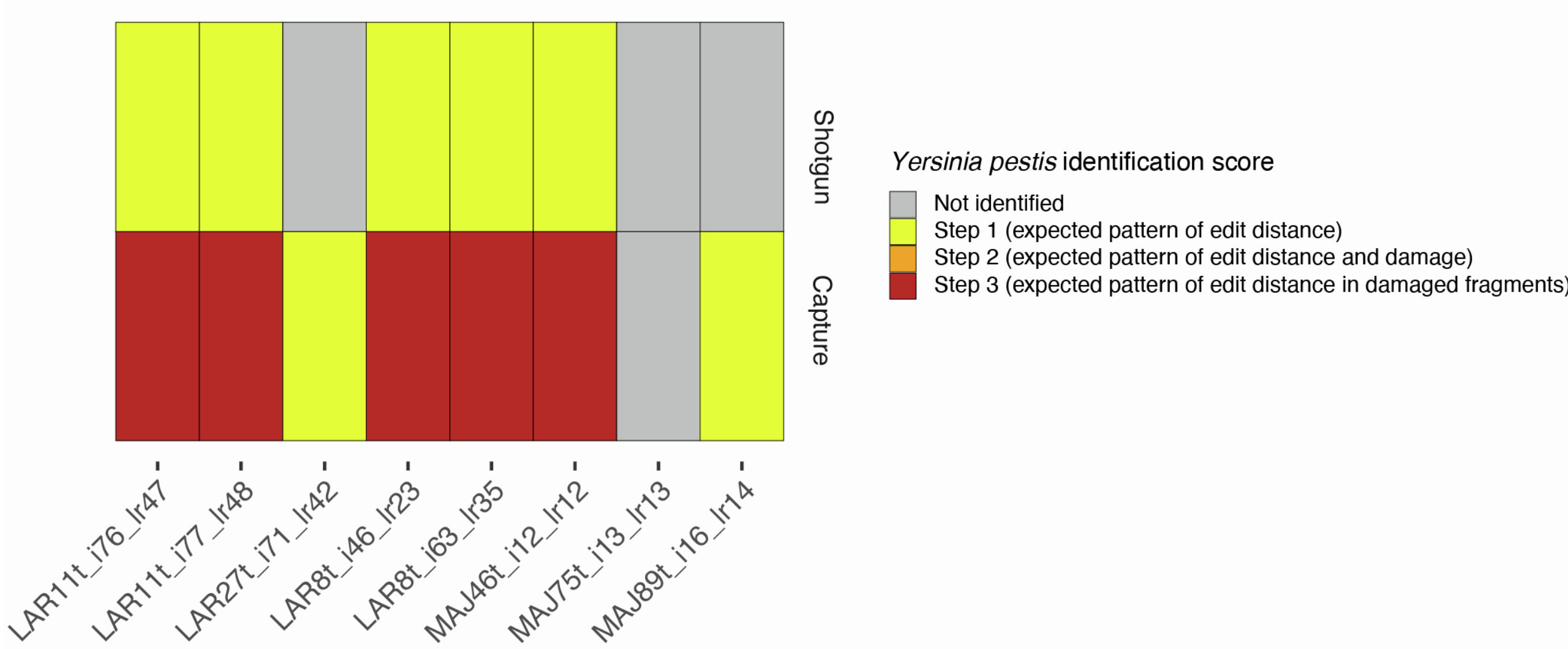

Figure S3: Results of *Yersinia pestis* detection using the *HOPS* ancient pathogen screening pipeline [S4]**.** All sequencing reads following filtering of human alignments were used as input. The results are highly similar to those obtained from *Y. pestis*-mapped reads (Fig. 4), with small differences: *Y. pestis* was not detected in one of the captured libraries (MAJ75t_i13_lr13), potentially due to non-specific mapping masking expected patterns of edit distance. The *Y. pestis* pathogen was, however, detected with higher scores (step 3) in two libraries (LAR8t_i63_lr35 and MAJ46t_i12_lr12).

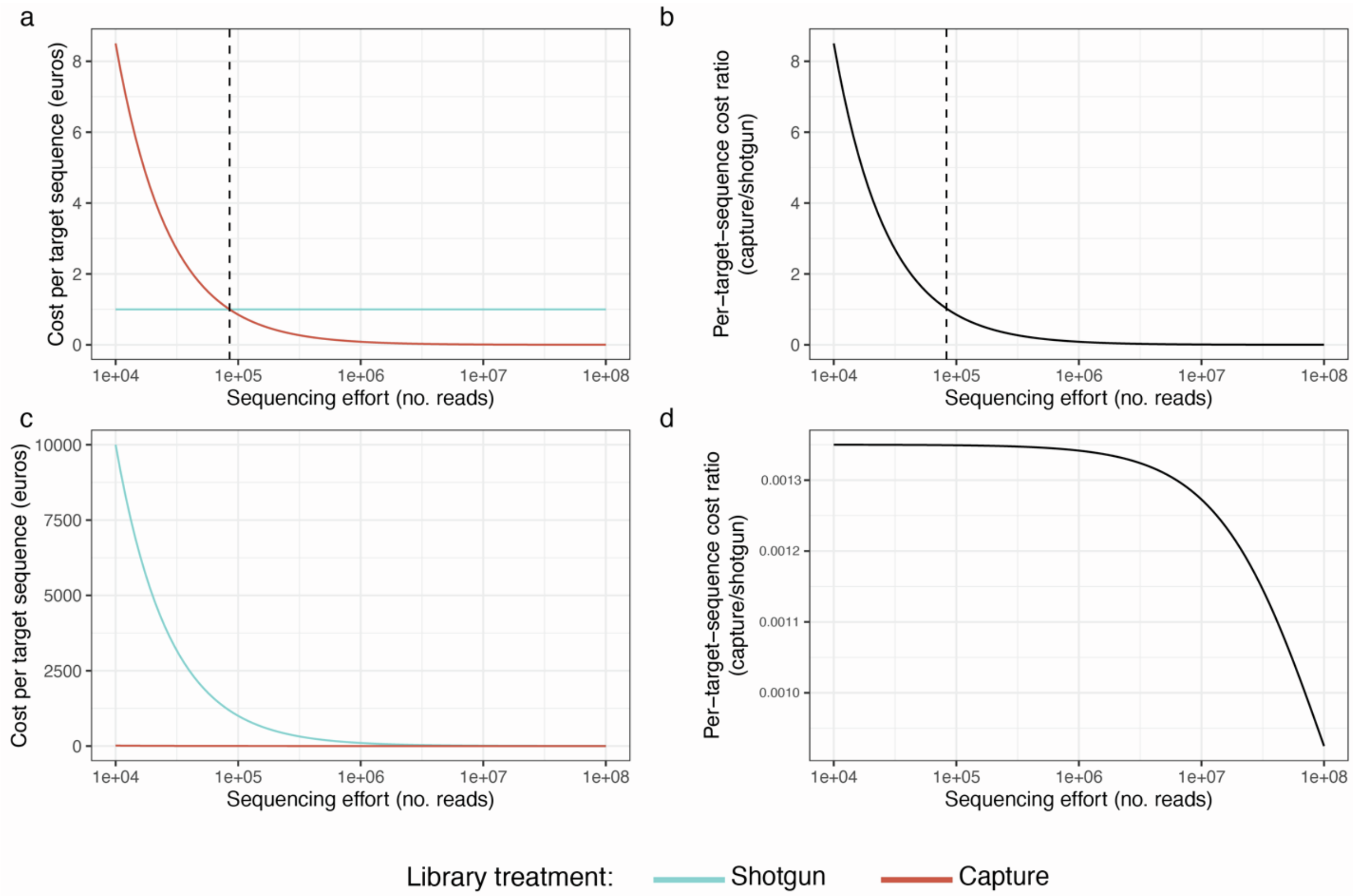

Figure S4: Comparison of cost per target pathogen sequence between shotgun sequencing and capture. The experiment cost is calculated as the product of sequencing effort and the cost per sequencing read in the case of shotgun sequencing (€1/M sequencing reads), plus the cost of library enrichment for capture (€170). Note that the respective costs are based on conservative estimates and that we do not account for the marginal costs related to potential amplification steps, sometimes also required for shotgun sequencing. In **(a)** and **(b)**, we excluded the cost associated with all previous laboratory steps, ranging from sampling to library preparation (corresponding to cases where the samples were initially processed for other aims than pathogen research). In **(c)** and **(d)**, we included these costs, estimated at €100, for both experiments (corresponding to cases where the samples were processed entirely for ancient pathogen research). The number of target pathogen sequences is calculated as the product of the sequencing effort and the fraction of target pathogen DNA in the library (fixed here to 1e-6; note that this parameter influences the absolute costs but has no influence on the cost ratio). The number of target pathogen sequences is further multiplied by an enrichment factor of 2,000 in the case of capture (the observed average value of targeted *Y. pestis* DNA enrichment in our test samples). The cost per target sequence is then obtained by dividing the number of target sequences by the respective experimental cost. **(a)** and **(c)** present the absolute cost per target sequence depending on sequencing effort for both strategies. **(b)** and **(d)** present the cost ratio of capture *vs*. shotgun sequencing depending on sequencing effort. The dotted vertical lines in **(a)** and **(b)** mark the sequencing effort above which capture becomes cost-effective. The benefit of capture relative to shotgun sequencing is always apparent when the costs of library preparation are included (**(c)** and **(d)**).

Method S1: Archaeological site description

The Lariey Cemetery (Puy Saint-Pierre, Hautes-Alpes) was excavated in 2002 as part of a planned archaeological investigation. This burial site is located in a wooded area on the outskirts of the three hamlets that form the village of Puy St-Pierre, approximately 5 km from Briançon.

Delineated in the landscape by rough stone block walls—visible today and recorded on the Napoleonic cadastral map—the cemetery lies at the edge of a clearing that likely corresponds to the site of an infirmary. However, no structural remains of the hospital have been found, suggesting the possible use of wooden huts or tents that may have been burned following the epidemic.

Based on four ^{14}C analyses and the study of ceramics found on-site, the cemetery is believed to date back to the early 17th century.

All of the individuals buried in this area were exhumed, totaling 34 bodies. Archaeological observations provide insight into the organization and function of the cemetery. Initial individual burials, likely representing the first deaths (in the northern part of the plot), were followed by double and simultaneous burials (in the central area), and eventually by multiple burials (in the southern part), reflecting a clear increase in mortality. Additional evidence supports the urgent nature of the burials in the southern section: some bodies were laid face down, insufficient sediment was used to cover the dead bodies, and stone blocks were employed to protect the remains from scavenging animals.

The creation and use of this cemetery for victims of the 1629–1630 epidemic is corroborated by historical archives, which report the presence of plague in 1630 throughout the Durance Valley, particularly in Pinet, one of the hamlets of Puy St-Pierre. However, it remains difficult to determine the precise demographic impact of the outbreak, especially the proportion of the local population represented by these 34 individuals buried.

Skeleton No. 8 (LAR8) belongs to a young adult female. Skeleton No. 11 (LAR11) belongs to an individual aged between 15 and 19 years, whose sex could not be determined. Skeleton No. 27 (LAR27) belongs to a young adult female.

The Mass Grave of La Major Cathedral (Marseille, Bouches-du-Rhône): the mass grave was discovered during a preventive excavation carried out in the autumn of 2008 in the eastern part of the esplanade of La Major Cathedral. This burial structure dates to the time of the 1720 plague

epidemic. Initially, the grave appeared in a zenithal view to be a south-north-oriented trench; however, this impression proved incorrect, as the excavated section (6 m by 1.5 m) represented only a small portion of a much larger burial pit. The full extent and boundaries of the pit could not be identified.

Despite this limitation, several archaeological observations leave no doubt regarding the historical context of this mortuary structure. Historical records corroborate this interpretation, providing evidence of the health crisis of 1720 and its demographic impact. City archives confirm the digging and use of mass graves in this area in September 1720, corresponding to the weeks of peak mortality in Marseille.

The large concentration of osteoarchaeological remains immediately struck the excavators. Numerous skeletons, some still articulated or anatomically consistent, were deposited in at least four discernible layers. The excavation revealed that the bodies had been deposited simultaneously, with a notable diversity in the orientation and position of the skeletons. This speaks to the urgency of the sanitary and funerary measures taken at the time.

Further evidence of this urgency comes from the artefacts found within the grave, some of which were directly associated with the bodies. These items suggest that the deceased were buried fully clothed. They included metal clothing accessories (such as shoe or belt buckles, and buttons), sometimes with remnants of organic material like leather, as well as small personal belongings, either worn by the victims or carried in their pockets. Among these was a coin dated 1720—a sixth of a silver Louis XV écu—which provides a *terminus post quem* for the grave.

By the end of the excavation, the remains of 106 more or less complete individuals had been recovered from this portion of the burial pit, including 81 adults and 25 subadults. Skeletons No. 46 (MAJ46) and No. 75 (MAJ75) belong to two adult females whose age could not be estimated osteologically. Skeleton No. 89 (MAJ89) belongs to a female over 40 years old.

Supplemental references

S1. Warinner, C., Herbig, A., Mann, A., Yates, J.A.F., Weiß, C.L., Burbano, H.A., Orlando, L., and Krause, J. (2017). A Robust Framework for Microbial Archaeology. Annual Review of Genomics and Human Genetics *18*, 321–356. https://doi.org/10.1146/annurev-genom-091416-035526.

S2. Jónsson, H., Ginolhac, A., Schubert, M., Johnson, P.L.F., and Orlando, L. (2013). mapDamage2.0: fast approximate Bayesian estimates of ancient DNA damage parameters. Bioinformatics *29*, 1682–1684. https://doi.org/10.1093/bioinformatics/btt193.

S3. Orlando, L., Allaby, R., Skoglund, P., Der Sarkissian, C., Stockhammer, P.W., Ávila-Arcos, M.C., Fu, Q., Krause, J., Willerslev, E., Stone, A.C., et al. (2021). Ancient DNA analysis. Nat Rev Methods Primers *1*, 1–26. https://doi.org/10.1038/s43586-020-00011-0.

S4. Hübler, R., Key, F.M., Warinner, C., Bos, K.I., Krause, J., and Herbig, A. (2019). HOPS: automated detection and authentication of pathogen DNA in archaeological remains. Genome Biology *20*, 280. https://doi.org/10.1186/s13059-019-1903-0.
